# Benchmarking hospital clinical pharmacy practice using standardised key performance indicators (KPIs)

**DOI:** 10.1080/20523211.2024.2431181

**Published:** 2024-12-16

**Authors:** Amelia R. Cossart, Martin L. Canning, Faith R. Yong, Christopher R. Freeman

**Affiliations:** a Safe and Effective Medicine Research Collaborative, Faculty of Medicine and Faculty of Health and Behavioural Science (School of Pharmacy), University of Queensland, Brisbane, Australia; b Metro North Clinical Governance, Herston, Australia; c Rural Clinical School, Faculty of Medicine, University of Queensland, Toowoomba, Australia

**Keywords:** Pharmacy, clinical pharmacy, KPI, medication safety, benchmark, patient safety, healthcare quality indicators

## Abstract

**Background:** Hospital pharmacy services support quality use of medicines and medication safety through clinical pharmacy activities such as medication reviews and patient education. These activities can be measured and monitored using evidence-based and standardised key performance indicators (KPIs), which highlight the value of pharmacy services. Standardisation of KPIs supports long-term benchmarking and inter- and intra-site comparisons to target key areas for improvement in clinical pharmacy services.

**Aim:** To describe the type and frequency of clinical pharmacy activity across five hospitals within one metropolitan hospital district.

**Methods:** Key Performance Indicator data were collected by pharmacists from five hospital sites at one metropolitan hospital district, in Queensland Australia. Data were collected over one week for the following clinical settings: inpatient, discharge, outpatient clinic, and the dispensary. Data were collected using a manual, paper-based data collection tool previously developed using a co-design process.

**Results:** Across 11,215 inpatient encounters, hospital pharmacy services provided: best possible medication history (BPMH) within 24 h of admission: 69.5%; daily medication chart review: 57.2%; discharge education: 82.7%, discharge reconciliation: 88.2%; and provision of discharge medication record: 82.4%. Across 1,092 outpatient encounters, pharmacists documented BPMH for 33.3% of patients. Pharmacists identified a total of 5,009 drug-related problems (DRPs) across the data collection period, with the rate of identification highest in the outpatient clinic setting (64.8 per 100 patient reviews) followed by discharge (52.6 per 100 patient reviews) and then inpatient (48.1 per 100 patient reviews). Almost 20% of DRPs identified (*n *= 975) were high risk.

**Conclusion:** Reporting and benchmarking clinical pharmacy activity through standardised KPIs supports opportunities to identify service improvements. Future research should focus on larger scale studies using routinely recorded data to monitor clinical pharmacy KPIs across all care settings.

## Background

Pharmacy services are crucial in supporting the quality use of medicines in the tertiary setting (Ng & Harrison, 2010). Hospital pharmacists conduct daily activities to optimise medication management and patient outcomes, including medication reviews, and education (for both patients and other healthcare professionals) (Teoh et al., 2017). Thus, pharmacists play a key role in reducing inappropriate prescribing, and in both identifying and resolving drug-related problems (DRPs) (Teoh et al., 2017). Quantification and benchmarking of pharmacy department performance is thus critical to demonstrate the effectiveness of medication safety processes, supporting the evaluation and improvement of clinical pharmacy service delivery.

The provision of patient-specific clinical pharmacy activities has demonstrated a range of improvements in both patient and health service usage outcomes, including reductions in patient mortality and medication error rates at transitions of care (Teoh et al., 2017). Improvements in health service usage include reductions in both readmissions and emergency department visits, resulting in cost savings (Canning, McDougall, et al., 2024; Daliri et al., 2021; Dooley et al., 2004; Mekonnen et al., 2016; Ravn-Nielsen et al., 2018; Scullin et al., 2007). Implementation of such evidence into practice has resulted in an increase in pharmacist services across different hospital settings in Australia (e.g. outpatient clinics), supporting medication safety for more patients (Canning et al., 2015; Snoswell et al., 2023; Snoswell et al., 2021). This increase in service provision has been encouraged by the Australian National Safety & Quality Health Service (NSQHS) Standards, specifically, NSQHS Medication Safety Standard (Standard Four) which mandates safe prescribing, dispensing, storing, manufacturing, and monitoring of medicines (Australian Commission on Safety and Quality in Health Care, 2021).

While the NSQHS Standards do not specifically define KPIs for pharmacy service activities, there is a requirement for health service organisations to demonstrate how they meet particular actions for reaccreditation, some of which align with clinical pharmacy service delivery (Australian Commission on Safety and Quality in Health Care, 2021). Clinical pharmacy activities such as best possible medication history documentation, medication reconciliation, medication review, provision of information to patients and generation of a current medicines list at transitions of care are evidence-based activities (Bond et al., 1999; Daliri et al., 2021; De Oliveira et al., 2021; Gillespie et al., 2009; Makowsky et al., 2009; Mekonnen et al., 2016; Ravn-Nielsen et al., 2018; Scullin et al., 2007; Stowasser et al., 2002) performed by pharmacists within our Health Service on a daily basis. However, across our hospitals, we have not had standardised definitions and data collection processes to enable monitoring and benchmarking. There is also limited benchmarking across Australia and indicators are currently open to interpretation (Carmichael et al., 2016). There has been some international reporting which proposes clinical pharmacy KPIs (Canning, Barras, et al., 2024; Fernandes et al., 2015; Lloyd et al., 2015, 2017; Lo et al., 2016; Ng & Harrison, 2010). However, there is a paucity of information which provides data on these KPIs to enable hospitals and pharmacy departments to benchmark performance internationally.

Meaningful measurement of performance is of critical importance to demonstrate the benefit of these services to overall hospital service delivery, to identify gaps in practice, and to advocate for additional human resources (Barbazza et al., 2021). In addition, collection and monitoring of clinical pharmacy KPIs, including descriptions and medications involved with drug-related problems identified by pharmacists, will assist in understanding where risks exist within medication management systems. Routine and ongoing collection will also help to understand how these risks may change over time.

## Method

### Aim

To quantify standardised clinical pharmacy activity KPIs across five hospitals within a single health service district. Specifically, we aimed to describe:

- The frequency and types of clinical activities performed by pharmacists.
- The frequency, risk, medicines and type of DRPs identified by pharmacists during routine activity.
- The differences in pharmacist service delivery between weekdays and weekends.

### Setting

This study was conducted at five hospitals within Metro North Hospital and Health Service (MNHHS) in 2022. MNHHS is the largest health service in Queensland, servicing a population of approximately 900,000 residents (Metro North Health, 2024). MNHHS provides a range of health services including acute care, rehabilitation, mental health and community care (Metro North Health, 2024). The types of hospitals included two tertiary referral hospitals with some state-wide quaternary services, a directorate providing community & subacute services, and two secondary hospitals (see Table 1).

**Table 1.** Participating sites.

Site	Site description	Approximate inpatient bed number
Site One	Regional hospital	290
Site Two	Metropolitan Community based services with subacute bedded services	120
Site Three	Regional hospital	250
Site Four	Metropolitan tertiary referral hospital with some quaternary services	986
Site Five	Metropolitan tertiary referral hospital with some quaternary services	690

### Data collection tool

The data collection tool used was co-designed and piloted with an expert working party of pharmacists across the health service (see Supplemental Material – Appendix). KPIs were aligned with the NSQHS actions for medication safety (Australian Commission on Safety and Quality in Health Care, 2021). The selection of these KPIs will be discussed in a separate published paper.

### Data collection and analysis

Data were recorded prospectively across one week (Monday – Sunday inclusive) during standard pharmacy service hours. Four sites recorded data from 25/7/22 to 31/7/22 and the fifth site recorded data from 1/8/22 to 7/8/22. All clinical pharmacists received locally organised training for the use of the data collection tool and recorded data as they went about their usual daily activities. This occurred across all hospital settings: inpatient (including discharge activities), outpatient clinics, and dispensaries.

The primary descriptive endpoints included the frequency and percentage of patients who received:

- best possible medication history (BPMH) within 24 h of admission,
- a medication (chart) review,
- medication-related education,
- discharge medicine reconciliation, and
- a discharge medication record (DMR) (also termed a medication list).

Additionally, pharmacists self-recorded the number of drug-related problems (DRPs) identified in accordance with a pre-determined classification list adapted from Basger et al. (Basger et al., 2015) These classifications are based on causes of drug-related errors, namely suboptimal prescribing, drug forms, dose, treatment duration, administration, logistics, monitoring, known adverse drug reactions and therapeutic advice. Pharmacists also risk-rated each DRP as ‘low’, ‘medium’, ‘high’ or ‘very high’ in accordance with the consequence and likelihood matrices in our Health Service Risk Management procedure (Allan, 2023) and the Society of Hospital Pharmacists of Australia (SHPA) Standards of Practice for Clinical Pharmacy Services (The Society of Hospital Pharmacists in Australia, 2013). Decision support was provided by embedding the consequence-likelihood-risk matrix, and example DRP classifications within the data collection tool. For ‘high’ or ‘very high’ risk ratings, pharmacists recorded a description of the DRP and the medicine involved.

An ethics exemption was obtained from MNHHS to collect data for hospital reporting and benchmarking purposes (ethics approval: EX/2022/MNHB/88484) which was ratified by the University of Queensland Research Ethics and Integrity body (2022/HE001566). Data was stored in a de-identified format, with any re-identifiable variables, including pharmacist and patient names, removed before analysis.

Data were entered into a Microsoft Excel™ spreadsheet, verified, cleaned and analysed using descriptive statistics, with frequency (percentages) or proportions (range, stratified by site) presented where appropriate.

## Results

In total, 11,215 inpatient and 1,092 outpatient occasions of service were evaluated. This included 2,021 newly admitted patients and 1,925 discharged patients. Pharmacists identified 5,009 drug-related problems (DRPs) across all care settings during the reporting period.

### Inpatient clinical pharmacy activities

During the study period, there were a total of ­­­11,215 inpatients across the 7 days. A total of 69.5% of new patients (*n *= 1,404) had a BPMH documented and verified within 24 h of admission, and 57.2% (*n *= 6,415) of all inpatients received a daily chart review (Table 2). Only 3.7% of patients (*n *= 411) received input from a pharmacist on a daily ward round, and 5.6% (*n *= 625) received daily inpatient education.

Pharmacists recorded a total of 3,084 (61.6%) DRPs in the inpatient setting, resulting in 48.1 interventions per 100 inpatient chart reviews. The most identified DRPs in the inpatient setting related to ‘medication omission’ (*n *= 574, 18.6%) followed by ‘therapeutic advice required for treatment’ (*n *= 412, 13.4%) and ‘dose too high’ (*n *= 215, 7.3%).

### Discharge activities

There was a total of 1,925 discharges across the 7 days (weekend-inclusive), of which 88.2% (*n *= 1,698) underwent discharge reconciliation and 82.4% (*n *= 1,587) were provided with a discharge medication record (DMR). The DMR included an accurate medication list and documented all medication changes during the inpatient stay. Only 4.1% of patients (*n *= 78) were referred for pharmacist-led optimisation post discharge, however, the majority (82.7%; *n *= 1,592) received verbal counselling and/or written information about their medicines prior to discharge. See Table 2 for demographic and KPI results by site.

Pharmacists recorded a total of 1,012 DRPs (20.2%) at discharge, resulting in an average of 52.6 interventions per 100 discharges. In the discharge setting, the most identified DRPs were ‘medication omission’ (*n *= 237, 23.4%) followed by ‘unclear or incomplete dosage instructions’ (*n *= 98, 9.7%) and ‘illegal, illegible or incomplete prescription order’ (*n *= 84; 8.3%).

### Outpatient clinic review

There was a total of 1,092 patients seen by a pharmacist in an outpatient clinic setting, representing 33.3% of clinic attendees. Of the patients seen by a pharmacist, 69.1% (*n *= 755) were provided medication education and 30.0% (*n *= 331) were provided a complete and accurate list of their current medications.

Overall, a total of 708 (14.1%) DRPs were identified by pharmacists in the clinic setting, which is an average of 64.8 interventions per 100 clinic patients seen. The most identified DRP was ‘therapeutic advice required for treatment’ (*n *= 277, 39.1%), ‘drug interactions’ (*n *= 100, 14.1%), and ‘disease state monitoring or therapeutic drug monitoring (TDM) required’ (*n *= 53, 7.4%).

### Dispensary

A total of 2,144 prescription sheets were dispensed across the 7 days. A prescription sheet can contain up to ten individual prescriptions for a patient. Only 1.1% (*n *= 24) of prescription sheets required modification by a dispensary pharmacist prior to dispensing (Table 2). From these prescription sheets, 205 (4.09%) DRPs were recorded resulting in an average of 9.6 interventions per 100 prescription sheets dispensed. The most identified DRPs related to ‘illegal, illegible, or incomplete prescription orders’ (*n *= 39, 19.0%) followed by ‘unclear/incomplete dosing instructions’ (*n *= 33, 16.1%) and the ‘prescribed drug not being available’ (*n *= 33, 16.1%).

### Site differences, workforce and weekends

There was variability in practice, service delivery and workforce staffing across the sites (Tables 2 and 3). When activities performed were normalised according to FTE, pharmacists had a daily average of 23.1 patients on their patient list, of which 4.2 were admitted within the previous 24 h, and 4 were discharged. Per day, each clinical pharmacist performed on average 2.9 admission BPMHs within 24 h, 13.2 medication (chart) reviews, 3.5 discharge reconciliations, and 3.3 patient education sessions (Table 4). When considering weekend activity, pharmacists had a higher average daily patient load (53.6 patients) which included more patients admitted within the previous 24 h (9.8 patients) and more discharges (6.1 patients) (Table 4).

**Table 2.** KPI clinical pharmacy services by site, presented as percentage of patients.

Service	Site 1 (Mon-Sun)	Site 2 (Mon-Sun)	Site 3 (Mon-Sun)	Site 4 (Mon-Sun)	Site 5 (Mon-Sun)	All (Mon-Sun)	All (Sat-Sun)
*Inpatients*							
Inpatient MAP within 24 h of arrival	57.3% (*n *= 125)	85.0% (*n *= 68)	75.7% (*n *= 318)	54.4% (*n *= 430)	90.4% (*n *= 463)	**69**.**5%**	57.5%
Daily inpatient chart review	32.4% (*n *= 401)	23.4% (*n *= 235)	65.9% (*n *= 959)	56.8% (*n *= 2321)	72.8% (*n *= 2499)	**57**.**2%**	22.9%
Daily ward round input by pharmacist	1.1% (*n *= 13)	2.3% (*n *= 23)	5.9% (*n *= 86)	5.6% (*n *= 229)	1.8% (*n *= 60)	**3**.**7%**	1.1%
Daily inpatient education	5.4% (*n *= 67)	4.1% (*n *= 41)	6.5% (*n *= 95)	5.2% (*n *= 212)	6.1% (*n *= 210)	**5**.**6%**	4.3%
Patients with discharge reconciliation	89.8% (*n *= 159)	78.7% (*n *= 48)	84.9% (*n *= 265)	85.0% (*n *= 801)	98.2% (*n *= 425)	**88**.**2%**	73.6%
Patients provided a DMR	87.0% (*n *= 154)	78.7% (*n *= 48)	82.1% (*n *= 256)	75.3% (*n *= 709)	97.0% (*n *= 420)	**82**.**4%**	56.6%
Patients provided discharge education	72.3% (*n *= 128)	44.3% (*n *= 27)	60.6% (*n *= 189)	88.8% (*n *= 836)	95.2% (*n *= 412)	**82**.**7%**	54.9%
Patients referred for pharmacist-led optimisation post discharge	4.5% (*n *= 8)	4.9% (*n *= 3)	4.8% (*n *= 15)	4.5% (*n *= 42)	2.3% (*n *= 10)	**4**.**1%**	2.6%
*Outpatient clinics*							
Medication history performed at clinic	44.2% (*n *= 46)	12.5% (*n *= 253)	4.6% (*n *= 5)	31.4% (*n *= 171)	60.8% (*n *= 306)	**33**.**3%**	12.0%
Clinical review at clinic	96.2% (*n *= 100)	13.9% (*n *= 281)	32.4% (*n *= 35)	34.9% (*n *= 190)	82.1% (*n *= 413)	**31**.**1%**	17.1%
Medication counselling by a pharmacist at clinic	28.9% (*n *= 30)	11.7% (*n *= 237)	100% (*n *= 108)	32.7% (*n *= 178)	40.2% (*n *= 202)	**23**.**0%**	11.8%
Collaborative prescribing at clinic	0.0% (*n *= 0)	0.2% (*n *= 3)	0.0% (*n *= 0)	0.6% (*n *= 3)	0.2% (*n *= 1)	**0**.**2%**	0.3%
Medication list provided at clinic	34.6% (*n *= 36)	2.4% (*n *= 49)	1.9% (*n *= 2)	22.0% (*n *= 120)	24.7% (*n *= 124)	**10**.**2%**	2.7%
*Outpatient dispensary*							
Prescription sheets with modifications	2.8% (*n *= 7)	N/A	0.4% (*n *= 2)	0.0% (*n *= 0)	1.1% (*n *= 15)	**1**.**1%**	0.7%

Note: n-value displayed in table represents numerator. Denominator for each metric is not displayed as this varies between site and indicator.

**Table 3.** The FTE pharmacists allocated to the inpatient, clinic, and dispensary settings, per site, per day.

	Monday	Tuesday	Wednesday	Thursday	Friday	Saturday	Sunday
*Site One*							
Inpatient	9.5	9.5	10	9.8	9	1	0
Clinic	0.5	0	1	1	0	0	0
Dispensary	1	1	1	1	1	1	1
*Site Two*							
Inpatient	12	13	12	13	10	1	0
Clinic	4	6	6	6	5	8	7
Dispensary	0	0	0	0	0	0	0
*Site Three*							
Inpatient	17	16	16	14	15	6	6
Clinic	15	14	14	12	13	5	5
Dispensary	2	2	2	2	2	1	1
*Site Four*							
Inpatient	27	26	24	28	28	9	4
Clinic	9	10	10	10	10	0	0
Dispensary	3	4	3	4	3	1	1
*Site Five*							
Inpatient	24	26	24	26	27.5	6.5	5
Clinic	7	6	5	6	4	0	0
Dispensary	4	4	4	4	4	1	1

**Table 4.** Average activities (services) delivered per pharmacist working in the inpatient setting.

	All days	Weekend only
Patients on list to see	23.13 (16.17–28.00)	53.56 (18.75–85.22)
New patients at admission	4.17 (1.31–5.42)	9.84 (6.00–12.77)
BPMH documented within 24 h of admission	2.90 (1.11–3.53)	5.66 (3.92–8.43)
Chart reviews during admission	13.23 (3.85–17.98)	12.29 (0–14.38)
Number of discharges	3.97 (1–6.45)	6.10 (3.00–10.92)
Discharge reconciliation	3.50 (0.79–5.49)	4.49 (2.33–7.85)
Discharge education	3.28 (0.44–5.73)	3.35 (1.00–7.00)

Note: Data presented as mean (range, stratified by site).

BPMH, best possible medication history.

### Drug-related problems

Pharmacists identified 5,009 drug-related problems (DRPs) across all care settings during the reporting period, of which 19.5% (*n *= 975) were regarded as high risk and 3.2% (*n *= 159) as very high risk (Table 5). The five main drugs causing high and very high-risk DRPs were nirmatrelvir/ritonavir (*n *= 66), enoxaparin (*n *= 63), rivaroxaban (*n *= 39), vancomycin (*n *= 36) and insulin (*n *= 32).

**Table 5.** Drug-related problems (DRPs), by site and location.

	Site 1	Site 2	Site 3	Site 4	Site 5	All sites
*Inpatient*						
Low	85 (36.17%)	88 (23.66%)	156 (24.26%)	82 (27.15%)	432 (28.2%)	843 (27.33%)
Medium	85 (36.17%)	161 (43.28%)	401 (62.36%)	138 (45.7%)	785 (51.24%)	1570 (50.91%)
High	62 (26.38%)	71 (19.09%)	75 (11.66%)	81 (26.82%)	293 (19.13%)	582 (18.87%)
Very high	3 (1.28%)	52 (13.98%)	11 (1.71%)	1 (0.33%)	22 (1.44%)	89 (2.89%)
*Discharge*						
Low	23 (27.38%)	4 (26.67%)	45 (16.42%)	70 (26.52%)	81 (21.6%)	223 (22.04%)
Medium	29 (34.52%)	9 (60%)	173 (63.14%)	129 (48.86%)	223 (59.47%)	563 (55.63%)
High	31 (36.9%)	2 (13.33%)	51 (18.61%)	64 (24.24%)	64 (17.07%)	212 (20.95%)
Very high	1 (1.19%)	0 (0%)	5 (1.82%)	1 (0.38%)	7 (1.87%)	14 (1.38%)
*Dispensary*						
Low	0 (0%)	0 (0%)	15 (46.88%)	80 (53.33%)	12 (80%)	107 (52.2%)
Medium	0 (0%)	0 (0%)	14 (43.75%)	44 (29.33%)	1 (6.67%)	59 (28.78%)
High	1 (12.5%)	0 (0%)	2 (6.25%)	25 (16.67%)	2 (13.33%)	30 (14.63%)
Very high	7 (87.5%)	0 (0%)	1 (3.13%)	1 (0.67%)	0 (0%)	9 (4.39%)
*Clinic*						
Low	8 (38.1%)	43 (33.86%)	12 (10%)	114 (47.3%)	56 (28.14%)	233 (32.91%)
Medium	3 (14.29%)	49 (38.58%)	57 (47.5%)	93 (38.59%)	75 (37.69%)	277 (39.12%)
High	3 (14.29%)	20 (15.75%)	31 (25.83%)	33 (13.69%)	64 (32.16%)	151 (21.33%)
Very high	7 (33.33%)	15 (11.81%)	20 (16.67%)	1 (0.41%)	4 (2.01%)	47 (6.64%)

Note: Data presented as number (percentage).

## Discussion

This study aimed to describe the type and frequency of pharmacist activity conducted across multiple hospitals and benchmark these metrics so that the impact of the clinical pharmacy activity on patient and medication safety could be better quantified. The results highlight that sites perform medication histories (69.5%), discharge reconciliation (88.2%), DMR provision (82.4%) and discharge education (82.7%) to a high proportion of inpatients.

This study highlights the variability in clinical pharmacy service delivery across five hospitals (Table 2). For example, the proportion of patients who had a medication history within 24 h of admission varied between 54% and 90% across the sites. During interpretation, it became apparent that the models of care used, and different clinical specialty services provided within different hospitals influence how useful KPI data was for benchmarking. For example, one site (Site 2) had a high proportion of patients receive a BPMH documented within 24 h of arrival, whereas a smaller proportion (23.41%) received a daily inpatient chart review. Site 2 offers community-based services and has some subacute inpatient bedded services; hence, service priorities would be to ensure a safe transition of care by documenting a BPMH at admission, however, daily clinical review of patients’ medicines may be less impactful due to the subacute nature of the patients. This contrasted with other sites, such as the two metropolitan hospitals (sites 4 and 5) whose patients are more likely to be acutely unwell, and correspondingly, had higher proportions of reported daily medication reviews by a pharmacist. Two sites also had higher rates of daily ward round input by a pharmacist, however, the absolute numbers and proportions of patients receiving pharmacy ward round input were relatively small. The evidence demonstrates some positive impact pharmacists can make through ward round attendance (Bullock et al., 2019; Bullock et al., 2020) and this is an opportunity for service expansion to improve the impact pharmacists have on optimising medicine outcomes for patients. Potentially, some sites have prioritised this activity over others, and this may explain their slightly higher rates for this activity.

This study also serves to highlight the differences in service delivery by clinical pharmacists on weekdays and weekends. With the exception of one site's clinic-based pharmacists on weekends, the number of pharmacist staff allocated to clinical areas on weekends across all sites was lower than on weekdays. This lower staffing on weekends may have influenced the KPI's collected as part of this study, since all documented weekend clinical activities were lower than weekday activities. These differences were particularly evident when the number of inpatients, newly admitted patients and discharge patients were normalised per FTE (Table 4), since allocated workload numbers were much lower on ‘all days’ when compared to weekends alone. Whilst this study did not collect data on patient categories to formally assess staffing against the Society of Hospital Pharmacists of Australia Standards of Practice (The Society of Hospital Pharmacists in Australia, 2013), these findings suggest that the patient list allocations are broadly aligned.

By reviewing the proportion of patients who received different clinical activities, assumptions can be made about how clinical activities were prioritised. On average, only 5.6% of patients had daily education provided by a pharmacist. This contrasts with 82.7% of patients who were provided education on discharge. This suggests that pharmacists prioritise the provision of education at discharge rather than daily during other clinical activities. While the aim of this study was not to capture the amount of time spent providing education, nor to assess the quality of education provision, future research should consider patient engagement and explore patient preference for when provision of medicines education occurs during the hospital stay.

For inpatients, it seems that sites prioritise discharge reconciliation, discharge education and provision of a DMR. This is evidenced when all days and weekends are considered separately, however, on weekends, BPMH for newly admitted patients seemed to be prioritised second to discharge reconciliation, and daily chart reviews were less of a priority. This prioritisation is supported by our findings which showed that pharmacists perform slightly higher interventions at discharge (52.57 interventions per 100 patient reviews) compared to admission and inpatient reviews (48.07 interventions per 100 patient reviews). A study within a similar geographical area stated hospital pharmacists prioritised patients with high-risk medicines, co-morbidities and admission reasons of higher acuity (Falconer et al., 2019), but did not explain task prioritisation. An integrated clinical pharmacy service has demonstrated improvements in patient flow (Tran et al., 2019) and poor patient flow leads to worse outcomes (Nguyen et al., 2022). As pharmacy discharge services are required before the patient can leave the hospital, supporting patient flow, may be one reason why these tasks are prioritised.

The rates of discharge reconciliation (88.2%) were higher than those for BPMH documentation within 24 h (69.5%). Ordinarily, discharge reconciliation could not occur without documentation of a BPMH. This study only measured BPMH recorded within 24 h of admission, as an aim of service delivery and a requirement of the NSQHS Standards is that a BPMH be documented on presentation or as early as possible, and the quality metric within 24 h of admission was used. It is likely, based on the discharge reconciliation rates, that the overall BPMH rates (regardless of timeframe since admission) are higher; however, this was not specifically recorded.

Pharmacists identified a high number of DRPs across the study period. The majority of DRPs were categorised by pharmacists as low- or medium-risk interventions. As part of the data collection process, pharmacists recorded the medicine involved with ‘high’ and ‘very high’ risk DRP's. The most commonly reported medicines causing high and very high-risk DRPs were also ‘high-risk’ medicines (Australian Commission on Safety and Quality in Health Care, 2021), which re-highlights the importance of pharmacy services in supporting medication safety and the quality use of medicines. The data collection period coincided with a peak COVID-19 wave, so this, along with the drug interactions associated with the combination COVID-19 therapeutic nirmatrelvir/ritonavir is the likely rationale why it was the medicine with the highest number of DRPs identified. Although the incidence of DRPs reported cannot be directly correlated to cost savings, as highlighted by Al-Jazairi and researchers in 2021, clinical pharmacists reduce hospital expenditure by directly altering patient care at the bedside through medication evaluation, before an adverse event occurs (Al-Jazairi & Alnakhli, 2021). Hence, collection and monitoring of medications associated with high-risk DRPs assists pharmacy departments to support service planning and expanded service delivery. It also enables monitoring of how medication-related risks may change over time.

The benefit of a pharmacist in the outpatient clinic has already been demonstrated, with Snoswell and researchers who conducted their study in a similarly sized metropolitan hospital district showing that 37% of patients had changes as a result of pharmacist intervention (Snoswell et al., 2021). This study was a preliminary study, with the implementation of pharmacists only a recent addition (Snoswell et al., 2021). Our study findings strengthen Snoswell’s findings and serve to highlight the importance of pharmacist activity to support patient-centred care and quality use of medicines. It could be argued that pharmacists had the highest impact within this clinical area as they performed the greatest number of clinical interventions (64.84 interventions per 100 patient reviews) in this setting. This is further supported by our finding that the number of DRPs identified in the dispensary was low. This may be because across the hospitals included in this study, the model of care prioritises clinical pharmacy activities on the ward, meaning that the majority of recorded interventions would be for ‘discharge’ prescriptions (as observed, there was a high number of discharge interventions) and the interventions made in the dispensary were likely related to outpatient prescriptions.

### Strengths and limitations

A strength of this study was the breadth of pharmacy services captured across five hospitals. By standardising the KPI definitions and collection across these hospitals through the use of one data collection tool, we have established its content validity and captured the extent of clinical pharmacist activity across hospital sites. This in turn enables comparative learnings and supports future benchmarking of services. Furthermore, standardisation of KPIs should enable routine measurement and evaluation of service across our health service district, which delineates priority care expectations, elevates pharmacist accountability and transparency, and importantly, supports quality use of medicines and patient safety, in line with the NSQHS Standards (Lo et al., 2016). Site-organised training for pharmacists about the tool prior to KPI collection further supported the consistency and rigour of the findings.

A drawback to these data was that the KPI measures collected as part of this study were process measures and do not measure outcomes (e.g. hospital readmission rates are a proxy for patient health). This means that it can be difficult to predict the true effect of pharmacist activity on patient outcomes. Further, patient descriptive statistics were unable to be collected. This was because the manual data collection process by pharmacists was cumbersome and potentially added to each pharmacist’s workload. Previously, this had been overcome by using routinely recorded data to measure the impact of the delivery of a ‘pharmaceutical care bundle’ on unplanned readmissions within 30 days (Canning, McDougall, et al., 2024). However, this care bundle did not measure the breadth of clinical activities, or the clinical settings in this study. This study builds upon those findings and provides benchmarks for other sites in other countries where clinical pharmacy services may still be developing. Such benchmarking may support increased funding for pharmacy services which is desirable as when collaboration between health professionals is nurtured, direct pharmacy involvement improves patient-centred care (Alefan & Halboup, 2016; Katoue et al., 2014; Lai et al., 2022; Said et al., 2020).

Although the data collected has given a descriptive overview of the activity conducted by pharmacists across five hospitals, data recording is likely to have been impacted by a peak COVID-19 wave which led to redistribution of the workforce during the data collection week. Our data was highly likely to have underestimated true activity per pharmacist, particularly as the tool was not designed to capture all pharmacist activity. Further, the study did not capture the role of pharmacy technicians and staff who support the pharmacy workforce and their valuable contribution, nor the effect of documentation of pharmacist activity itself on data collection. As explained by Minard and researchers in 2016 (Minard et al., 2016), pharmacists find KPI collection challenging due to the increased workload and documentation requirements, environment constraints, and balancing competing priorities, which likely impacted our findings. Studies report that pharmacists tend not to document simple interventions or routine tasks performed on a daily basis (Al-Jazairi & Alnakhli, 2021; Ng & Harrison, 2010), making it difficult to accurately quantify the true scope of pharmacist activity. Despite this limitation, use of a similar methodology would be useful for benchmarking purposes and future automated digital capture of these interventions has the potential to improve data collection.

The data collected focused on process measures and the extent of activity delivered only, not the quality of service provided. Whilst the collection of information relating to DRPs provides some evidence of quality, it is unknown whether all DRPs for a patient were identified and resolved. Similarly, the quality of data collection may have varied between pharmacists and between sites. However, standardised data collection tools and training were used to support uniformity of data collection and assist in overcoming these limitations.

### Future directions

Continued collection of KPI data at specified time intervals and the introduction of automated electronic data collection is crucial to support the expansion and refinement of clinical pharmacy services, and thus ensure pharmacy activities delivered support patient and medication safety, upholding the NSQHS Standards. By automating the data collection processes using routinely recorded data, the administrative burden on pharmacists will be reduced (Lai et al., 2022) and it will ensure the data collected is more concordant with true activity.

## Conclusion

This study has demonstrated that standardised clinical pharmacy activity KPIs can be quantified across five hospitals within a single health service district to enable benchmarking. Over a seven-day period almost 70% of inpatients received the best possible medication history documented by a pharmacist within 24 h of admission and almost 90% of patients received discharge reconciliation. The rate of drug-related problem identification varied across clinical settings and almost half of DRPs were risk-rated as ‘medium risk’. Future research should repeat this study on a larger scale (e.g. state-wide collection) and use routinely recorded data to monitor clinical pharmacy KPIs across all care settings.

**List of Abbreviations**
DRP: drug-related problems
KPI: key performance indicator
NSQHS : National Safety & Quality Health Service

## Supplementary Material

Supplemental Material - Appendix
