# Supplementary material for: Benchmarking hospital clinical pharmacy practice using standardised key performance indicators (KPIs)
Source: J Pharm Policy Pract. 2024 Dec 16;17(1):2431181. doi: 10.1080/20523211.2024.2431181 (PMC11650437; doi:10.1080/20523211.2024.2431181)
Supplement: Supplemental Material - Appendix [file JPPP_A_2431181_SM8206.pdf]

❑ Caboolture ❑ Sunshine
❑ COH ❑ G ❑ N
❑ RBWH ❑ STARS
❑ Redcliffe ❑ TPCH

PHARMACY INPATIENT KPI COLLECTION SHEET

Team/Ward	Shift (e.g. 9am-5pm)	Date

Pharmacist	

Team/ward activities

Please fill in the numbers of:

Total patients on list to see today	New pts admitted to facility	MAPs done within ≤24 hours	Charts reviewed today	Ward round patients with input

Patient education provided

Please fill in the numbers of:

SEE TABLE A FOR APINCHS CATEGORIES

Total patients provided education	Patients provided APINCHS medication education	Patients provided non-APINCHS medication education	Pts. provided other education (e.g. devices, antibiotic infusors, lifestyle)

Drug-related problems (DRPs)

Please additionally document APINCHS, high and very high interventions on the back.

FOR EXAMPLE:
L LOW RISK INTERVENTION
H *TRIANGLE FOR* **WARD ROUND DRP**

CODE	Description	Risk rating - *see table B* L= low, M = medium, H= high, VH= very high
1.2	**Drug prescribing** No indication for drug	
1.1	Inappropriate drug (C/I, not optimal drug)	
1.3	Interaction present	
1.4	Indication not treated	
1.5	More cost-effective drug available	
1.6	Omission (medication not charted)	
1.7	Duplication (drug doubled up)	
2.1	**Drug form** inappropriate/suboptimal	
3.1	**Dose** Too low	
3.2	Too high	
3.3	Not frequent enough	
3.4	Too frequent	
3.6	Unclear/incomplete dosage instructions	
4.1	**Treatment duration** Duration too short	
4.2	Duration too long	

CODE	Description	Risk rating - *see table B* L= low, M =medium, H= high, VH= very high
5.4	**Administration** Drug not administered	
5.1	Inappropriate administration timing/dosing intervals	
5.2	Underadministration of drug	
5.3	Overadministration of drug	
5.5	Wrong drug selected from shelf, taken or administered	
5.6	Drug abused	
5.7	Drug/form not used as directed	
5.8	Administration information needed to be given	
5.9	Inappropriate storage	
6.1	**Logistics** Error in drug selection	
6.2	Drug unavailable (non-LAM/ PBS, OOS)	
6.3	Illegal, illegible or incomplete Rx order	
7.1	**Monitoring:** disease state monitoring or TDM required	
7.3	Inappropriate test ordered	
8.1	**Known ADR** flagged, incl. allergy/previous ADR	
9.1	**Therapeutic advice** provided for treatment	

PHARMACY INPATIENT KPI COLLECTION SHEET:
APINCHS, HIGH & VERY HIGH RISK INCIDENTS

Drug-related problems (DRPs) description

Patient UR	Medication/s involved (if any)	Risk rating	Intervention code	Description

KPI Cheatsheet Tables

Table A: APINCHS medication

Category	Medication example
A: Antimicrobials	Aminoglycosides: gentamicin, tobramycin and amikacin<br>Vancomycin<br>Amphotericin – liposomal formulation
P: Potassium and other electrolytes	Injections of concentrated electrolytes: potassium, magnesium, calcium, hypertonic sodium chloride
I: Insulin	All insulins
N: Narcotics (opioids) other sedatives	Hydromorphone, oxycodone, morphine, fentanyl, alfentanil, remifentanil and analgesic patches<br>Benzodiazepines: diazepam, midazolam<br>Thiopentone, propofol and other short term anaesthetics
C: Chemotherapy agents	Vincristine, methotrexate, etoposide, azathioprine<br>Oral chemotherapy
H: Heparin and other anticoagulants	Heparin and low molecular weight heparins (LMWH): dalteparin, enoxaparin<br>Warfarin<br>Direct oral anticoagulants (DOACs): dabigatran, rivaroxaban, apixaban
S: Systems	Medication safety systems such as independent double checks, safe administration of liquid medications, standardised order sets, medication charts

Table B: MNHHS Risk Rating (Low to Very High)

1. RATE THE LIKELIHOOD
2. RATE THE CONSEQUENCE
3. FIND WHERE THE LIKELIHOOD AND CONSEQUENCE MEET ON THE TABLE TO FIND THE RATING (LOW, MEDIUM, HIGH, VERY HIGH)
4. PUT RISK RATING ON DRP INTERVENTION TABLE IN THE RELEVANT CATEGORY

TABLE B FIELDS	QUESTION BEING ASKED
LIKELIHOOD	How likely is it that the clinical harm (this harm is from the patient's perspective) you're addressing would happen? The assumption is that the risk will not be identified by other staff during LOS or post-discharge evidence-based treatment duration.
CONSEQUENCE	How serious is the clinical risk you are addressing, in the worst case scenario?

LIKELIHOOD	CONSEQUENCE: NEGLIGIBLE	MINOR	MODERATE	MAJOR	EXTREME
ALMOST CERTAIN	Medium	Medium	High	Very High	Very High
LIKELY	Medium	Medium	High	High	Very High
POSSIBLE	Low	Medium	High	High	High
UNLIKELY	Low	Medium	Medium	Medium	High
RARE	Low	Low	Low	Medium	High

Risk severity (clinical)

Category	Severity for preventable clinical incidents (Health Service Delivery)	
B1. Negligible	No harm.	SAC3
B2. Minor	Minimal preventable harm. First aid treatment only	SAC3
B3. Moderate	Temporary preventable harm.	SAC2
B4. Major	Permanent preventable harm/loss of function/disability	SAC1
B5. Extreme	Preventable loss of life	SAC1

Risk frequency (clinical)

Likelihood	**Frequency -** *event expected to occur:*
A1. Almost certain (>90%)	Once every week or month
A2. Likely (60-90%)	Once every 1-2 years
A3. Possible (30-60%)	Once every 5 years
A4. Unlikely (5-30%)	Once every 10 years
A5. Rare	Once in more than 10 years

Inpatient KPI cheatsheet

Team/ward activities

FIELD	DEFINITION
PATIENTS ON LIST TO SEE TODAY	The number of patients that you are allocated for the day
NEW PATIENTS TO FACILITY	Total new patients to facility admitted yesterday and today that are your ward/team's responsibility. *(This is for calculating which patients have been seen in the last 24 hours.)*
MAPS DONE WITHIN ≤24 HOURS	If the patient was admitted yesterday, count this if the MAP (or PAHN for iEMR sites) is completed by close of business (COB) today. MAP/PAHN may have been finalised in ED, transfer ward or by yourself.
CHARTS REVIEWED TODAY	The total number of patient charts reviewed in the day, including admission and discharge chart reviews.
WARD ROUND PATIENTS WITH INPUT	Patients you gave input into while on the ward round / multidisciplinary team where medication decisions are made.

Patient education

FIELD	DEFINITION
TOTAL PATIENTS PROVIDED EDUCATION	Total number of patients you interacted with to teach information about their medication, disease state or lifestyle. If education is provided for an **inpatient**, record here. *Discharge counselling is documented on the discharge sheet, NOT this section.*
PATIENTS PROVIDED APINCHS MEDICATION EDUCATION	The number of patients you interacted with to teach them information about an APINCHS medication (see Table A), including related disease state counselling and/or lifestyle changes required.
PATIENTS PROVIDED NON-APINCHS MEDICATION EDUCATION	All other patient interactions involving medication education that was non-APINCHS (see Table A).
PATIENTS PROVIDED OTHER EDUCATION (E.G. DEVICES, ANTIBIOTIC INFUSERS, LIFESTYLE ONLY)	Patient interactions where you taught them about using medical devices or lifestyle changes required.

Risk rating & DRP examples	CONSEQUENCE	PROBABILITY	OVERALL RISK	DRP
VTE prophylaxis not prescribed. Outcome of intervention: Charted	EXTREME	POSSIBLE	**HIGH**	1.6
IHD, Patient charted metoprolol when previous ADR existed (depression) team notified. Outcome: Ceased	MODERATE	LIKELY	**HIGH**	8.1
Dose of gliclazide recently increased, patient has poor liver function secondary to alcoholism, at risk of hypoglycaemia. Outcome: Dose reduced	MODERATE	UNLIKELY	**MEDIUM**	3.2
On discharge suggest potassium return to usual dose as potassium level increasing. Dose on d/c script higher than usual dose. Outcome: Dose changed	MODERATE	POSSIBLE	**HIGH**	3.2
Medications charted for wrong patient – medications included Targin 40/20 *bd* in elderly opioid naive patient with poor lungs. Identified before patient received medications. Outcome: Medications charted for correct patient	EXTREME	LIKELY	**VERY HIGH**	1.1<br>1.6
Patient charted incorrect dose of Parkinson's medications, may have caused dyskinesia's/falls.	MAJOR	UNLIKELY	**MEDIUM**	3.2
Amiloride not charted. Outcome: Dr charted	MODERATE	UNLIKELY	**MEDIUM**	3.2
Patient had NSTEM - no anti-coagulation charted. Outcome: Charted heparin infusion	MAJOR	ALMOST CERTAIN	**VERY HIGH**	1.6
Charted spironolactone 25mg morning. Pre-admission dose 50mg bd for diuresis. Outcome: Dose changed to usual dose	MODERATE	LIKELY	**HIGH**	3.1,<br>3.3
Taking paracetamol 665mg at home. Not on formulary. Outcome: Changed to paracetamol 500mg	NEGLIGIBLE	RARE	**LOW**	6.2
Patient with ischaemic heart disease and LDL 3.3 on atorvastatin 20mg daily. Outcome: Dose increased to 80mg	EXTREME	UNLIKELY	**HIGH**	3.1
Oxybutynin not charted. Outcome: RMO added.	MINOR	POSSIBLE	**MEDIUM**	1.6
Prn oral oxycodone, no paracetamol order. Outcome: Regular paracetamol charted	MINOR	UNLIKELY	**MEDIUM**	1.4
Non LAM: fosinopril swapped to Ramipril	NEGLIGIBLE	RARE	**LOW**	6.2
Isosorbide mononitrate doses *bd*. May cause tolerance. Outcome: Changed to daily dose	MODERATE	LIKELY	**HIGH**	3.2
Patient taking simvastatin 40mg, IHD. Started on voriconazole 200mg bd. Outcome: Dr withheld simvastatin while on voriconazole to prevent rhabdo.	EXTREME	POSSIBLE	**HIGH**	1.3
Atorvastatin 40mg charted, usual dose 10mg. Unintentional error. Outcome: Dr changed to usual dose	MINOR	RARE	**LOW**	3.2

PHARMACY DISCHARGE KPI COLLECTION SHEET

- ❑ Caboolture ❑ Sunshine
- ❑ COH ❑ G ❑ N
- ❑ RBWH ❑ STARS
- ❑ Redcliffe ❑ TPCH

Team/Ward	Shift (e.g. 9am-5pm)	Date

Pharmacist	

Discharge activities

Please fill in the numbers of:

Total patients discharged		Number of external liaisons	
Total pts w/ pharmacist D/C rec.		Pts. for whom DAA was organised	
DMRs/med. lists provided		Pts. D/C without review	
Pts. referred to pharmacist-led d/c optimisation			

Patients provided education:

Total	
APINCHS	
Non-APINCHS	
Other (devices, lifestyle...)	

Reasons for patient discharge without pharmacist review

Out of hours		Nurse-initiated		Pt self D/C		Poor staffing		Ph. deemed pt. as low risk	
Other		OTHER REASONS							

Drug-related problems (DRPs)

Please additionally document APINCHS, high and very high interventions on the back.

FOR EXAMPLE:
L LOW RISK INTERVENTION
H *TRIANGLE FOR* **WARD ROUND DRP**

CODE	Description	Risk rating - *see table B* L= low, M = medium, H= high, VH= very high	CODE	Description	Risk rating - *see table B* L= low, M =medium, H= high, VH= very high
1.2	**Drug prescribing** No indication for drug		5.4	**Administration** Drug not administered	
1.1	Inappropriate drug (C/I, not optimal drug)		5.1	Inappropriate administration timing/dosing intervals	
1.3	Interaction present		5.2	Underadministration of drug	
1.4	Indication not treated		5.3	Overadministration of drug	
1.5	More cost-effective drug available		5.5	Wrong drug selected from shelf, taken or administered	
1.6	Omission (medication not charted)		5.6	Drug abused	
1.7	Duplication (drug doubled up)		5.7	Drug/form not used as directed	
2.1	**Drug form** inappropriate/suboptimal		5.8	Administration information needed to be given	
3.1	**Dose** Too low		5.9	Inappropriate storage	
3.2	Too high		6.1	**Logistics** Error in drug selection	
3.3	Not frequent enough		6.2	Drug unavailable (non-LAM/ PBS, OOS)	
3.4	Too frequent		6.3	Illegal, illegible or incomplete Rx order	
3.6	Unclear/incomplete dosage instructions		7.1	**Monitoring:** disease state monitoring or TDM required	
4.1	**Treatment duration** Duration too short		7.3	Inappropriate test ordered	
4.2	Duration too long		8.1	**Known ADR** flagged, incl. allergy/previous ADR	
			9.1	**Therapeutic advice** provided for treatment	

PHARMACY DISCHARGE KPI COLLECTION SHEET:
APINCHS, HIGH & VERY HIGH RISK INCIDENTS

Drug-related problems (DRPs) description

Patient UR	Medication/s involved (if any)	Risk rating	Intervention code	Description

KPI Cheatsheet Tables

Table A: APINCHS medication

Category	Medication example
A: Antimicrobials	Aminoglycosides: gentamicin, tobramycin and amikacin<br>Vancomycin<br>Amphotericin – liposomal formulation
P: Potassium and other electrolytes	Injections of concentrated electrolytes: potassium, magnesium, calcium, hypertonic sodium chloride
I: Insulin	All insulins
N: Narcotics (opioids) other sedatives	Hydromorphone, oxycodone, morphine, fentanyl, alfentanil, remifentanil and analgesic patches<br>Benzodiazepines: diazepam, midazolam<br>Thiopentone, propofol and other short term anaesthetics
C: Chemotherapy agents	Vincristine, methotrexate, etoposide, azathioprine<br>Oral chemotherapy
H: Heparin and other anticoagulants	Heparin and low molecular weight heparins (LMWH): dalteparin, enoxaparin<br>Warfarin<br>Direct oral anticoagulants (DOACs): dabigatran, rivaroxaban, apixaban
S: Systems	Medication safety systems such as independent double checks, safe administration of liquid medications, standardised order sets, medication charts

Table B: MNHHS Risk Rating (Low to Very High)

1. RATE THE LIKELIHOOD
2. RATE THE CONSEQUENCE
3. FIND WHERE THE LIKELIHOOD AND CONSEQUENCE MEET ON THE TABLE TO FIND THE RATING (LOW, MEDIUM, HIGH, VERY HIGH)
4. PUT RISK RATING ON DRP INTERVENTION TABLE IN THE RELEVANT CATEGORY

TABLE B FIELDS	QUESTION BEING ASKED
LIKELIHOOD	How likely is it that the clinical harm (this harm is from the patient's perspective) you're addressing would happen? The assumption is that the risk will not be identified by other staff during LOS or post-discharge evidence-based treatment duration.
CONSEQUENCE	How serious is the clinical risk you are addressing, in the worst case scenario?

LIKELIHOOD	CONSEQUENCE: NEGLIGIBLE	MINOR	MODERATE	MAJOR	EXTREME
ALMOST CERTAIN	Medium	Medium	High	Very High	Very High
LIKELY	Medium	Medium	High	High	Very High
POSSIBLE	Low	Medium	High	High	High
UNLIKELY	Low	Medium	Medium	Medium	High
RARE	Low	Low	Low	Medium	High

Risk severity (clinical)

Category	Severity for preventable clinical incidents (Health Service Delivery)	
B1. Negligible	No harm.	SAC3
B2. Minor	Minimal preventable harm. First aid treatment only	SAC3
B3. Moderate	Temporary preventable harm.	SAC2
B4. Major	Permanent preventable harm/loss of function/disability	SAC1
B5. Extreme	Preventable loss of life	SAC1

Risk frequency (clinical)

Likelihood	**Frequency -** *event expected to occur:*
A1. Almost certain (>90%)	Once every week or month
A2. Likely (60-90%)	Once every 1-2 years
A3. Possible (30-60%)	Once every 5 years
A4. Unlikely (5-30%)	Once every 10 years
A5. Rare	Once in more than 10 years

Discharge KPI cheatsheet

Discharge activities

FIELD	DEFINITION
TOTAL PATIENTS DISCHARGED	Total daily number of patients allocated to the ward/team who were discharged from hospital.
TOTAL PATIENTS W/ PHARMACIST D/C REC.	Total number of patients whose discharge medications have been reconciled by a pharmacist
DMRS/ MEDICATION LISTS PROVIDED	The number of patients to whom DMRs/medication lists were provided
PATIENTS COUNSELLED VERBALLY	How many patients the pharmacist interacted with verbally to provide medication counselling on discharge. Do not include if only written information (digital or paper) was provided.
NUMBER OF EXTERNAL LIAISONS	Significant enquiry to external professional (e.g. PACS, HITH, RACF, QOTP prescriber, GP practice, disability support worker, paid carer) to transfer specific patient information or coordinate patient care.<br>Do not include DAA changes and organisation. Counselling family member/unpaid carer would be counted as patient education.
PTS. REFERRED TO PHARMACIST-LED D/C OPTIMISATION	When patients are referred for pharmacist-led discharge optimisation such as medication reviews, including HMRs and similar services
PTS. FOR WHOM DAA WAS ORGANISED	Patients for whom Webster packs, dosettes and other dose administration aids are organised by the pharmacist, through contacting community pharmacy, GP, carer and other relevant personnel. If other information is provided outside of DAA organisation, record that as a separate intervention.
PTS. D/C WITHOUT REVIEW	The number of patients who were discharged without pharmacist review.

Reasons for patient discharge without pharmacist review

FIELD	DEFINITION
OUT OF HOURS	Patient was discharged when there was no pharmacist present to perform a review (out of hours, meal/toilet break)
NURSE-INITIATED	When the nurse initiates patient discharge without alerting other health professionals.
PT SELF D/C	The patient discharged themselves from the ward.
POOR STAFFING	Low levels of pharmacist staff resulted in some patients not receiving a pharmacist review before their discharge.
PH. DEEMED PT. AS LOW RISK	The pharmacist assessed the patient as low risk compared to other patients present, and thus did not perform a pharmacist review before discharge.
OTHER	A different reason to the above caused patient discharge without pharmacist review. If this is chosen, the reason should be entered in the “other reasons” field.

Patient education

WORKED EXAMPLE: COUNSELLING A PATIENT ON A NEW PRESCRIPTION FOR TARGIN & PARACETAMOL, PLUS HEAT PACKS/EXERCISE WOULD COUNT AS: APINCHS: 1, NON-APINCHS: 1, OTHER: 1

FIELD	DEFINITION
TOTAL PATIENTS PROVIDED EDUCATION	Total number of patients you interacted with to teach information about their medication, disease state or lifestyle. If **discharge counselling** is provided for an inpatient, record here. *Inpatient counselling is documented on the inpatient sheet, NOT this section.*
PATIENTS PROVIDED APINCHS MEDICATION EDUCATION	The number of patients you interacted with to teach them information about an APINCHS medication (see Table A), including related disease state counselling and/or lifestyle changes required.
PATIENTS PROVIDED NON-APINCHS MEDICATION EDUCATION	All other patient interactions involving medication education that was non-APINCHS (see Table A).
PATIENTS PROVIDED OTHER EDUCATION (E.G. DEVICES, ABX INFUSERS, LIFESTYLE ONLY)	Patient interactions where you taught them about using medical devices or lifestyle changes required.

Risk rating & DRP examples	CONSEQUENCE	PROBABILITY	OVERALL RISK	DRP
VTE prophylaxis not prescribed. Outcome of intervention: Charted	EXTREME	POSSIBLE	**HIGH**	1.6
IHD, Patient charted metoprolol when previous ADR existed (depression) team notified. Outcome: Ceased	MODERATE	LIKELY	**HIGH**	8.1
Dose of gliclazide recently increased, patient has poor liver function secondary to alcoholism, at risk of hypoglycaemia. Outcome: Dose reduced	MODERATE	UNLIKELY	**MEDIUM**	3.2
On discharge suggest potassium return to usual dose as potassium level increasing. Dose on d/c script higher than usual dose. Outcome: Dose changed	MODERATE	POSSIBLE	**HIGH**	3.2
Medications charted for wrong patient – medications included Targin 40/20 *bd* in elderly opioid naive patient with poor lungs. Identified before patient received medications. Outcome: Medications charted for correct patient	EXTREME	LIKELY	**VERY HIGH**	1.1<br>1.6
Patient charted incorrect dose of Parkinson’s medications, may have caused dyskinesia’s/falls.	MAJOR	UNLIKELY	**MEDIUM**	3.2
Amiloride not charted. Outcome: Dr charted	MODERATE	UNLIKELY	**MEDIUM**	3.2
Patient had NSTEM - no anti-coagulation charted.<br>Outcome: Charted heparin infusion	MAJOR	ALMOST CERTAIN	**VERY HIGH**	1.6
Charted spironolactone 25mg morning. Pre-admission dose 50mg bd for diuresis. Outcome: Dose changed to usual dose	MODERATE	LIKELY	**HIGH**	3.1,<br>3.3

ED - PHARMACY KPI COLLECTION SHEET

- ❑ Caboolture
- ❑ COH
- ❑ RBWH
- ❑ Redcliffe
- ❑ Sunshine
  - ❑ G ❑ N
- ❑ STARS
- ❑ TPCH

Shift (e.g. 9am-5pm)	Pharmacist	Date

Pharmacy ED activities

*Please fill in the numbers of **patients** who received:*

Total pts. seen	Pharmacist history only	Medication history on MAP form	Medication reconciliations	Pharmacist clinical review	ADR identification & management	Resus. assistance
**Medical consult.**	**Nurse/ allied health consult.**	**Collaborative prescribing**	**Medication counselling**	**Prescription reviews**	**Discharge Medication Record**	**External liaisons**

Drug-related problems (DRPs)

Please additionally document APINCHS, high and very high interventions on the back.

FOR EXAMPLE:
L LOW RISK INTERVENTION
H *TRIANGLE FOR* WARD ROUND DRP

CODE	Description	Risk rating - *see table B* L= low, M = medium, H= high, VH= very high
1.2	**Drug prescribing** No indication for drug	
1.1	Inappropriate drug (C/I, not optimal drug)	
1.3	Interaction present	
1.4	Indication not treated	
1.5	More cost-effective drug available	
1.6	Omission (medication not charted)	
1.7	Duplication (drug doubled up)	
2.1	**Drug form** inappropriate/suboptimal	
3.1	**Dose** Too low	
3.2	Too high	
3.3	Not frequent enough	
3.4	Too frequent	
3.6	Unclear/incomplete dosage instructions	
4.1	**Treatment duration** Duration too short	
4.2	Duration too long	

CODE	Description	Risk rating - *see table B* L= low, M =medium, H= high, VH= very high
5.4	**Administration** Drug not administered	
5.1	Inappropriate administration timing/dosing intervals	
5.2	Underadministration of drug	
5.3	Overadministration of drug	
5.5	Wrong drug selected from shelf, taken or administered	
5.6	Drug abused	
5.7	Drug/form not used as directed	
5.8	Administration information needed to be given	
5.9	Inappropriate storage	
6.1	**Logistics** Error in drug selection	
6.2	Drug unavailable (non-LAM/ PBS, OOS)	
6.3	Illegal, illegible or incomplete Rx order	
7.1	**Monitoring:** disease state monitoring or TDM required	
7.3	Inappropriate test ordered	
8.1	**Known ADR** flagged, incl. allergy/previous ADR	
9.1	**Therapeutic advice** provided for treatment	

PHARMACY ED KPI COLLECTION SHEET:
APINCHS, HIGH & VERY HIGH RISK INCIDENTS

Drug-related problems (DRPs) description

Patient UR	Medication/s involved (if any)	Risk rating	Intervention code	Description

KPI Cheatsheet Tables

Table A: APINCHS medication

Category	Medication example
A: Antimicrobials	Aminoglycosides: gentamicin, tobramycin and amikacin<br>Vancomycin<br>Amphotericin – liposomal formulation
P: Potassium and other electrolytes	Injections of concentrated electrolytes: potassium, magnesium, calcium, hypertonic sodium chloride
I: Insulin	All insulins
N: Narcotics (opioids) other sedatives	Hydromorphone, oxycodone, morphine, fentanyl, alfentanil, remifentanil and analgesic patches<br>Benzodiazepines: diazepam, midazolam<br>Thiopentone, propofol and other short term anaesthetics
C: Chemotherapy agents	Vincristine, methotrexate, etoposide, azathioprine<br>Oral chemotherapy
H: Heparin and other anticoagulants	Heparin and low molecular weight heparins (LMWH): dalteparin, enoxaparin<br>Warfarin<br>Direct oral anticoagulants (DOACs): dabigatran, rivaroxaban, apixaban
S: Systems	Medication safety systems such as independent double checks, safe administration of liquid medications, standardised order sets, medication charts

Table B: MNHHS Risk Rating (Low to Very High)

1. RATE THE LIKELIHOOD
2. RATE THE CONSEQUENCE
3. FIND WHERE THE LIKELIHOOD AND CONSEQUENCE MEET ON THE TABLE TO FIND THE RATING (LOW, MEDIUM, HIGH, VERY HIGH)
4. PUT RISK RATING ON DRP INTERVENTION TABLE IN THE RELEVANT CATEGORY

TABLE B FIELDS	QUESTION BEING ASKED
LIKELIHOOD	How likely is it that the clinical harm (this harm is from the patient's perspective) you're addressing would happen? The assumption is that the risk will not be identified by other staff during LOS or post-discharge evidence-based treatment duration.
CONSEQUENCE	How serious is the clinical risk you are addressing, in the worst case scenario?

		CONSEQUENCE				
		NEGLIGIBLE	MINOR	MODERATE	MAJOR	EXTREME
LIKELIHOOD	ALMOST CERTAIN	Medium	Medium	High	Very High	Very High
	LIKELY	Medium	Medium	High	High	Very High
	POSSIBLE	Low	Medium	High	High	High
	UNLIKELY	Low	Medium	Medium	Medium	High
	RARE	Low	Low	Low	Medium	High

Risk severity (clinical)

Category	Severity for preventable clinical incidents (Health Service Delivery)	
B1. Negligible	No harm.	SAC3
B2. Minor	Minimal preventable harm. First aid treatment only	SAC3
B3. Moderate	Temporary preventable harm.	SAC2
B4. Major	Permanent preventable harm/loss of function/disability	SAC1
B5. Extreme	Preventable loss of life	SAC1

Risk frequency (clinical)

Likelihood	**Frequency -** *event expected to occur:*
A1. Almost certain (>90%)	Once every week or month
A2. Likely (60-90%)	Once every 1-2 years
A3. Possible (30-60%)	Once every 5 years
A4. Unlikely (5-30%)	Once every 10 years
A5. Rare	Once in more than 10 years

ED KPI cheatsheet

FIELD	DEFINITION
TOTAL PTS. SEEN	The number of patients the pharmacist has attended to (whether through their documentation/records or in person) in the ED for the day.
PHARMACIST HISTORY ONLY	Medication history only: BPMHx documented on medication chart or in EDIS notes.
MEDICATION HISTORY ON MAP FORM	Medication history documented on the patient's MAP form.
MEDICATION RECONCILIATION	Reconciliation on MAP form<br>Medication chart annotated
PHARMACIST CLINICAL REVIEW	Clinical review of patient with or without MAP<br>OR Review acute management of patient in the ED<br>OR Ensuring on-the-dot medications prescribed and supplied
ADR IDENTIFICATION & MANAGEMENT	New ADR – documented , reported and managed appropriately<br>OR Conducting allergy history/investigation<br>Do not include if standard allergy/adverse reaction history is taken as part of medication history
RESUS. ASSISTANCE	Assistance with medication scribing, medication choice/dose and/or preparation/reconstitution of medications in a resuscitation/trauma
MEDICAL CONSULT	Number of requests by ED MO for either medication enquiry, consult and/or recommendation. Do not include if pharmacist refers patients to ED MO.
NURSE/ALLIED HEALTH CONSULT	Request by ED nurse/allied health staff (e.g. DABIT/social worker/physio) for medication related enquiry, consult and/or recommendation. Do not include if pharmacist refers patients to ED nurse/allied health staff.
COLLABORATIVE PRESCRIBING	Pharmacist prescribing with MO countersign for ED patients. Do not record when no doctor countersigns medication recommendation.
MEDICATION COUNSELLING	Face-to-face interaction where medication use is explained, including treatment regimen etc. to patient and/or carer
PRESCRIPTION REVIEW	Pharmacist review of prescription written by Dr, before the prescription is given to patient<br>OR Clinical +/- clerical review
DISCHARGE MEDICATION RECORD	Provision of a medication list to patient/carer (DMR/iMAR/EDDMAR)
EXTERNAL LIAISONS	Significant enquiry to external professional (e.g. RACF, QOTP prescriber, GP practice) to obtain specific patient information, or arrange DAA changes. Not to be used for standard confirmation of medicines as part of medication history

**Risk rating & DRP examples**	CONSEQUENCE	PROBABILITY	OVERALL RISK	DRP
VTE prophylaxis not prescribed. Outcome of intervention: Charted	EXTREME	POSSIBLE	**HIGH**	1.6
IHD, Patient charted metoprolol when previous ADR existed (depression) team notified. Outcome: Ceased	MODERATE	LIKELY	**HIGH**	8.1
Dose of gliclazide recently increased, patient has poor liver function secondary to alcoholism, at risk of hypoglycaemia. Outcome: Dose reduced	MODERATE	UNLIKELY	**MEDIUM**	3.2
On discharge suggest potassium return to usual dose as potassium level increasing. Dose on d/c script higher than usual dose. Outcome: Dose changed	MODERATE	POSSIBLE	**HIGH**	3.2
Medications charted for wrong patient – medications included Targin 40/20 *bd* in elderly opioid naive patient with poor lungs. Identified before patient received medications. Outcome: Medications charted for correct patient	EXTREME	LIKELY	**VERY HIGH**	1.1<br>1.6
Patient charted incorrect dose of Parkinson's medications, may have caused dyskinesia's/falls.	MAJOR	UNLIKELY	**MEDIUM**	3.2
Amiloride not charted. Outcome: Dr charted	MODERATE	UNLIKELY	**MEDIUM**	3.2
Patient had NSTEM - no anti-coagulation charted.<br>Outcome: Charted heparin infusion	MAJOR	ALMOST CERTAIN	**VERY HIGH**	1.6
Charted spironolactone 25mg morning. Pre-admission dose 50mg bd for diuresis.<br>Outcome: Dose changed to usual dose	MODERATE	LIKELY	**HIGH**	3.1,<br>3.3
Taking paracetamol 665mg at home. Not on formulary. Outcome: Changed to paracetamol 500mg	NEGLIGIBLE	RARE	**LOW**	6.2
Patient with ischaemic heart disease and LDL 3.3 on atorvastatin 20mg daily.<br>Outcome: Dose increased to 80mg	EXTREME	UNLIKELY	**HIGH**	3.1
Oxybutynin not charted. Outcome: RMO added.	MINOR	POSSIBLE	**MEDIUM**	1.6
Prn oral oxycodone, no paracetamol order.<br>Outcome: Regular paracetamol charted	MINOR	UNLIKELY	**MEDIUM**	1.4

❑ Caboolture ❑ Sunshine
❑ COH ❑ G ❑ N
❑ RBWH ❑ STARS
❑ Redcliffe ❑ TPCH

CLINIC - PHARMACY KPI COLLECTION SHEET

Clinic	Pharmacist	Date

Clinic activities

Please tally the numbers of:

P = PHONE CONSULT, F: FACE TO FACE, T = TELEHEALTH

	Total pts. at clinic	Total patients seen	Medication history	Pharmacist clinical review	Pts' Pathology checked	Medical consults	Nurse/ allied health consults
P							
F							
T							

	Medication counselling	Collaborative prescribing patients	Prescription reviews only	Medication lists provided	DAAs organised	External liaisons
P						
F						
T						

NB: ALL PROTOCOL-BASED RECOMMENDATIONS COUNT AS INTERVENTIONS

Drug-related problems (DRPs)

Please additionally document APINCHS, high and very high interventions on the back.

FOR EXAMPLE:
L LOW RISK INTERVENTION
H *TRIANGLE FOR* **WARD ROUND DRP**

CODE	Description	Risk rating - *see table B* L= low, M = medium, H= high, VH= very high	CODE	Description	Risk rating - *see table B* L= low, M =medium, H= high, VH= very high
1.2	**Drug prescribing** No indication for drug		5.4	**Administration** Drug not administered	
1.1	Inappropriate drug (C/I, not optimal drug)		5.1	Inappropriate administration timing/dosing intervals	
1.3	Interaction present		5.2	Underadministration of drug	
1.4	Indication not treated		5.3	Overadministration of drug	
1.5	More cost-effective drug available		5.5	Wrong drug selected from shelf, taken or administered	
1.6	Omission (medication not charted)		5.6	Drug abused	
1.7	Duplication (drug doubled up)		5.7	Drug/form not used as directed	
2.1	**Drug form** inappropriate/suboptimal		5.8	Administration information needed to be given	
3.1	**Dose** Too low		5.9	Inappropriate storage	
3.2	Too high		6.1	**Logistics** Error in drug selection	
3.3	Not frequent enough		6.2	Drug unavailable (non-LAM/ PBS, OOS)	
3.4	Too frequent		6.3	Illegal, illegible or incomplete Rx order	
3.6	Unclear/incomplete dosage instructions		7.1	**Monitoring:** disease state monitoring or TDM required	
4.1	**Treatment duration** Duration too short		7.3	Inappropriate test ordered	
4.2	Duration too long		8.1	**Known ADR** flagged, incl. allergy/previous ADR	
			9.1	**Therapeutic advice** provided for treatment	

PHARMACY CLINIC KPI COLLECTION SHEET:
APINCHS, HIGH & VERY HIGH RISK INCIDENTS

Drug-related problems (DRPs) description

Patient UR	Medication/s involved (if any)	Risk rating	Intervention code	Description

KPI Cheatsheet Tables

Table A: APINCHS medication

Category	Medication example
A: Antimicrobials	Aminoglycosides: gentamicin, tobramycin and amikacin<br>Vancomycin<br>Amphotericin – liposomal formulation
P: Potassium and other electrolytes	Injections of concentrated electrolytes: potassium, magnesium, calcium, hypertonic sodium chloride
I: Insulin	All insulins
N: Narcotics (opioids) other sedatives	Hydromorphone, oxycodone, morphine, fentanyl, alfentanil, remifentanil and analgesic patches<br>Benzodiazepines: diazepam, midazolam<br>Thiopentone, propofol and other short term anaesthetics
C: Chemotherapy agents	Vincristine, methotrexate, etoposide, azathioprine<br>Oral chemotherapy
H: Heparin and other anticoagulants	Heparin and low molecular weight heparins (LMWH): dalteparin, enoxaparin<br>Warfarin<br>Direct oral anticoagulants (DOACs): dabigatran, rivaroxaban, apixaban
S: Systems	Medication safety systems such as independent double checks, safe administration of liquid medications, standardised order sets, medication charts

Table B: MNHHS Risk Rating (Low to Very High)

1. RATE THE LIKELIHOOD
2. RATE THE CONSEQUENCE
3. FIND WHERE THE LIKELIHOOD AND CONSEQUENCE MEET ON THE TABLE TO FIND THE RATING (LOW, MEDIUM, HIGH, VERY HIGH)
4. PUT RISK RATING ON DRP INTERVENTION TABLE IN THE RELEVANT CATEGORY

TABLE B FIELDS	QUESTION BEING ASKED
LIKELIHOOD	How likely is it that the clinical harm (this harm is from the patient's perspective) you're addressing would happen? The assumption is that the risk will not be identified by other staff during LOS or post-discharge evidence-based treatment duration.
CONSEQUENCE	How serious is the clinical risk you are addressing, in the worst case scenario?

LIKELIHOOD \ CONSEQUENCE	NEGLIGIBLE	MINOR	MODERATE	MAJOR	EXTREME
ALMOST CERTAIN	Medium	Medium	High	Very High	Very High
LIKELY	Medium	Medium	High	High	Very High
POSSIBLE	Low	Medium	High	High	High
UNLIKELY	Low	Medium	Medium	Medium	High
RARE	Low	Low	Low	Medium	High

Risk severity (clinical)

Category	Severity for preventable clinical incidents (Health Service Delivery)	
B1. Negligible	No harm.	SAC3
B2. Minor	Minimal preventable harm. First aid treatment only	SAC3
B3. Moderate	Temporary preventable harm.	SAC2
B4. Major	Permanent preventable harm/loss of function/disability	SAC1
B5. Extreme	Preventable loss of life	SAC1

Risk frequency (clinical)

Likelihood	Frequency - *event expected to occur:*
A1. Almost certain (>90%)	Once every week or month
A2. Likely (60-90%)	Once every 1-2 years
A3. Possible (30-60%)	Once every 5 years
A4. Unlikely (5-30%)	Once every 10 years
A5. Rare	Once in more than 10 years

Clinic KPI cheatsheet

FIELD	DEFINITION
TOTAL PTS AT CLINIC	How many patients attended the clinic in that day. Do not include patients who did not attend clinic.
TOTAL PTS SEEN	How many patients the pharmacist interacted with, or had intervened in their treatment, during the day
MEDICATION HISTORY	Medication history documented within clinical documentation
PHARMACIST CLINICAL REVIEW	Clinical review of patient with or without BPMH: this should be an assessment of medication treatment appropriateness
PTS' PATHOLOGY CHECKED	The pharmacist checked pathology results to see if treatment was appropriate for the patient.
EXTERNAL LIAISONS	Significant enquiry to external professional (e.g. PACS, HITH, RACF, QOTP prescriber, GP practice, disability support worker, paid carer) to obtain specific patient information. Can be used for standard confirmation of medicines as part of medication history
MEDICAL CONSULTS	Number of requests by medical officers for either medication enquiry, consult and/or recommendations.
NURSE/ALLIED HEALTH CONSULTS	Number of requests nurse/allied health staff) for medication related enquiry, consult and/or recommendations.
COLLABORATIVE PRESCRIBING PATIENTS	Pharmacist prescribing with MO countersign for admission orders and outpatient prescriptions
MEDICATION COUNSELLING	How many patients the pharmacist interacted with verbally to provide medication counselling.
PRESCRIPTION REVIEWS	Pharmacist review of outpatient prescriptions written by prescribers (I.e. including Clinical +/- clerical review)
MEDICATION LISTS PROVIDED	The number of patients to whom DMRs/medication lists were provided
DAAS ORGANISED	Patients for whom Webster packs, dosettes and other dose administration aids are organised by the pharmacist, through contacting community pharmacy, GP, carer and other relevant personnel.

Risk rating & DRP examples	CONSEQUENCE	PROBABILITY	OVERALL RISK	DRP
VTE prophylaxis not prescribed. Outcome of intervention: Charted	EXTREME	POSSIBLE	**HIGH**	1.6
IHD, Patient charted metoprolol when previous ADR existed (depression) team notified. Outcome: Ceased	MODERATE	LIKELY	**HIGH**	8.1
Dose of gliclazide recently increased, patient has poor liver function secondary to alcoholism, at risk of hypoglycaemia. Outcome: Dose reduced	MODERATE	UNLIKELY	**MEDIUM**	3.2
On discharge suggest potassium return to usual dose as potassium level increasing. Dose on d/c script higher than usual dose. Outcome: Dose changed	MODERATE	POSSIBLE	**HIGH**	3.2
Medications charted for wrong patient – medications included Targin 40/20 *bd* in elderly opioid naive patient with poor lungs. Identified before patient received medications. Outcome: Medications charted for correct patient	EXTREME	LIKELY	**VERY HIGH**	1.1<br>1.6
Patient charted incorrect dose of Parkinson's medications, may have caused dyskinesia's/falls.	MAJOR	UNLIKELY	**MEDIUM**	3.2
Amiloride not charted. Outcome: Dr charted	MODERATE	UNLIKELY	**MEDIUM**	3.2
Patient had NSTEM - no anti-coagulation charted.<br>Outcome: Charted heparin infusion	MAJOR	ALMOST CERTAIN	**VERY HIGH**	1.6
Charted spironolactone 25mg morning. Pre-admission dose 50mg bd for diuresis.<br>Outcome: Dose changed to usual dose	MODERATE	LIKELY	**HIGH**	3.1,<br>3.3
Taking paracetamol 665mg at home. Not on formulary. Outcome: Changed to paracetamol 500mg	NEGLIGIBLE	RARE	**LOW**	6.2
Patient with ischaemic heart disease and LDL 3.3 on atorvastatin 20mg daily.<br>Outcome: Dose increased to 80mg	EXTREME	UNLIKELY	**HIGH**	3.1
Oxybutynin not charted. Outcome: RMO added.	MINOR	POSSIBLE	**MEDIUM**	1.6
Prn oral oxycodone, no paracetamol order.<br>Outcome: Regular paracetamol charted	MINOR	UNLIKELY	**MEDIUM**	1.4
Non LAM: fosinopril swapped to Ramipril	NEGLIGIBLE	RARE	**LOW**	6.2
Isosorbide mononitrate doses *bd*. May cause tolerance.<br>Outcome: Changed to daily dose	MODERATE	LIKELY	**HIGH**	3.2
Patient taking simvastatin 40mg, IHD. Started on voriconazole 200mg bd.<br>Outcome: Dr withheld simvastatin while on voriconazole to prevent rhabdo.	EXTREME	POSSIBLE	**HIGH**	1.3
Atorvastatin 40mg charted, usual dose 10mg. Unintentional error.<br>Outcome: Dr changed to usual dose	MINOR	RARE	**LOW**	3.2

❑ Caboolture ❑ Sunshine
❑ COH ❑ G ❑ N
❑ RBWH ❑ STARS
❑ Redcliffe ❑ TPCH

OUTPATIENT DISPENSARY - PHARMACY KPI SHEET

Team/Ward	Pharmacist	Date

Prescriptions dispensed

	Outpatient	Discharge	Leave	Other	Monitored meds.
Total Rx sheets dispensed					
Total Rx sheets with modification					

Patient education provided

SEE TABLE A FOR APINCHS CATEGORIES

	Outpatient	Discharge	Leave	Other
Total patients				
APINCHS patients				
Non-APINCHS patients				
Other (devices, lifestyle)				

Drug-related problems (DRPs)

FOR EXAMPLE: L LOW RISK INTERVENTION

H *TRIANGLE FOR* **WARD ROUND DRP**

Please additionally document APINCHS, high and very high interventions on the back.

CODE	Description	Risk rating - *see table B* L= low, M = medium, H= high, VH= very high	CODE	Description	Risk rating - *see table B* L= low, M =medium, H= high, VH= very high
1.2	**Drug prescribing** No indication for drug		5.4	**Administration** Drug not administered	
1.1	Inappropriate drug (C/I, not optimal drug)		5.1	Inappropriate administration timing/dosing intervals	
1.3	Interaction present		5.2	Underadministration of drug	
1.4	Indication not treated		5.3	Overadministration of drug	
1.5	More cost-effective drug available		5.5	Wrong drug selected from shelf, taken or administered	
1.6	Omission (medication not charted)		5.6	Drug abused	
1.7	Duplication (drug doubled up)		5.7	Drug/form not used as directed	
2.1	**Drug form** inappropriate/suboptimal		5.8	Administration information needed to be given	
3.1	**Dose** Too low		5.9	Inappropriate storage	
3.2	Too high		6.1	**Logistics** Error in drug selection	
3.3	Not frequent enough		6.2	Drug unavailable (non-LAM/ PBS, OOS)	
3.4	Too frequent		6.3	Illegal, illegible or incomplete Rx order	
3.6	Unclear/incomplete dosage instructions		7.1	**Monitoring:** disease state monitoring or TDM required	
4.1	**Treatment duration** Duration too short		7.3	Inappropriate test ordered	
4.2	Duration too long		8.1	**Known ADR** flagged, incl. allergy/previous ADR	
			9.1	**Therapeutic advice** provided for treatment	

OUTPATIENT DISPENSARY KPI COLLECTION SHEET:
APINCHS, HIGH & VERY HIGH RISK INCIDENTS

Drug-related problems (DRPs) description

Patient UR	Medication/s involved (if any)	Risk rating	Intervention code	Description

KPI Cheatsheet Tables

Table A: APINCHS medication

Category	Medication example
A: Antimicrobials	Aminoglycosides: gentamicin, tobramycin and amikacin<br>Vancomycin<br>Amphotericin – liposomal formulation
P: Potassium and other electrolytes	Injections of concentrated electrolytes: potassium, magnesium, calcium, hypertonic sodium chloride
I: Insulin	All insulins
N: Narcotics (opioids) other sedatives	Hydromorphone, oxycodone, morphine, fentanyl, alfentanil, remifentanil and analgesic patches<br>Benzodiazepines: diazepam, midazolam<br>Thiopentone, propofol and other short term anaesthetics
C: Chemotherapy agents	Vincristine, methotrexate, etoposide, azathioprine<br>Oral chemotherapy
H: Heparin and other anticoagulants	Heparin and low molecular weight heparins (LMWH): dalteparin, enoxaparin<br>Warfarin<br>Direct oral anticoagulants (DOACs): dabigatran, rivaroxaban, apixaban
S: Systems	Medication safety systems such as independent double checks, safe administration of liquid medications, standardised order sets, medication charts

Table B: MNHHS Risk Rating (Low to Very High)

1. RATE THE LIKELIHOOD
2. RATE THE CONSEQUENCE
3. FIND WHERE THE LIKELIHOOD AND CONSEQUENCE MEET ON THE TABLE TO FIND THE RATING (LOW, MEDIUM, HIGH, VERY HIGH)
4. PUT RISK RATING ON DRP INTERVENTION TABLE IN THE RELEVANT CATEGORY

TABLE B FIELDS	QUESTION BEING ASKED
LIKELIHOOD	How likely is it that the clinical harm (this harm is from the patient's perspective) you're addressing would happen? The assumption is that the risk will not be identified by other staff during LOS or post-discharge evidence-based treatment duration.
CONSEQUENCE	How serious is the clinical risk you are addressing, in the worst case scenario?

LIKELIHOOD	CONSEQUENCE: NEGLIGIBLE	MINOR	MODERATE	MAJOR	EXTREME
ALMOST CERTAIN	Medium	Medium	High	Very High	Very High
LIKELY	Medium	Medium	High	High	Very High
POSSIBLE	Low	Medium	High	High	High
UNLIKELY	Low	Medium	Medium	Medium	High
RARE	Low	Low	Low	Medium	High

Risk severity (clinical)

Category	Severity for preventable clinical incidents (Health Service Delivery)	
B1. Negligible	No harm.	SAC3
B2. Minor	Minimal preventable harm. First aid treatment only	SAC3
B3. Moderate	Temporary preventable harm.	SAC2
B4. Major	Permanent preventable harm/loss of function/disability	SAC1
B5. Extreme	Preventable loss of life	SAC1

Risk frequency (clinical)

Likelihood	**Frequency -** *event expected to occur:*
A1. Almost certain (>90%)	Once every week or month
A2. Likely (60-90%)	Once every 1-2 years
A3. Possible (30-60%)	Once every 5 years
A4. Unlikely (5-30%)	Once every 10 years
A5. Rare	Once in more than 10 years

Outpatient Dispensary KPI cheatsheet

Prescriptions dispensed

FIELD	DEFINITION
TOTAL RX SHEETS DISPENSED	The number of pieces of paper prescriptions are printed on which have been dispensed
TOTAL RX SHEETS WITH MODIFICATION	The number of sheets paper prescriptions are on which have been modified and dispensed
OUTPATIENT	Patients who are not admitted to the hospital
DISCHARGE	Prescriptions for patients being discharged from the hospital
LEAVE	Prescriptions for patients needing leave medication
OTHER	Any other prescriptions for patients
MONITORED MED. ITEMS	Controlled substances and schedule 8 medication items

WORKED EXAMPLE: COUNSELLING A PATIENT ON A NEW PRESCRIPTION FOR TARGIN & PARACETAMOL, PLUS HEAT PACKS/EXERCISE WOULD COUNT AS: (APINCHS: 1, NON-APINCHS: 1, OTHER: 1)

Patient education

FIELD	DEFINITION
TOTAL PATIENTS PROVIDED EDUCATION	Total number of patients you interacted with to teach information about their medication, disease state or lifestyle. (If you have provided a patient discharge counselling this is documented on the inpatient and discharge form)
PATIENTS PROVIDED APINCHS MEDICATION EDUCATION	The number of patients you interacted with to teach them information about an APINCHS medication (see Table A), including related disease state counselling and/or lifestyle changes required.
PATIENTS PROVIDED NON-APINCHS MEDICATION EDUCATION	All other patient interactions involving medication education that was non-APINCHS (see Table A).
PATIENTS PROVIDED OTHER EDUCATION (E.G. DEVICES, ANTIBIOTIC INFUSERS, LIFESTYLE)	Patient interactions where you taught them about using medical devices or lifestyle changes required.

Risk rating & DRP examples	CONSEQUENCE	PROBABILITY	OVERALL RISK	DRP
VTE prophylaxis not prescribed. Outcome of intervention: Charted	EXTREME	POSSIBLE	**HIGH**	1.6
IHD, Patient charted metoprolol when previous ADR existed (depression) team notified. Outcome: Ceased	MODERATE	LIKELY	**HIGH**	8.1
Dose of gliclazide recently increased, patient has poor liver function secondary to alcoholism, at risk of hypoglycaemia. Outcome: Dose reduced	MODERATE	UNLIKELY	**MEDIUM**	3.2
On discharge suggest potassium return to usual dose as potassium level increasing. Dose on d/c script higher than usual dose. Outcome: Dose changed	MODERATE	POSSIBLE	**HIGH**	3.2
Medications charted for wrong patient – medications included Targin 40/20 *bd* in elderly opioid naive patient with poor lungs. Identified before patient received medications. Outcome: Medications charted for correct patient	EXTREME	LIKELY	**VERY HIGH**	1.1<br>1.6
Patient charted incorrect dose of Parkinson's medications, may have caused dyskinesia's/falls.	MAJOR	UNLIKELY	**MEDIUM**	3.2
Amiloride not charted. Outcome: Dr charted	MODERATE	UNLIKELY	**MEDIUM**	3.2
Patient had NSTEM - no anti-coagulation charted.<br>Outcome: Charted heparin infusion	MAJOR	ALMOST CERTAIN	**VERY HIGH**	1.6
Charted spironolactone 25mg morning. Pre-admission dose 50mg bd for diuresis.<br>Outcome: Dose changed to usual dose	MODERATE	LIKELY	**HIGH**	3.1,<br>3.3
Taking paracetamol 665mg at home. Not on formulary. Outcome: Changed to paracetamol 500mg	NEGLIGIBLE	RARE	**LOW**	6.2
Patient with ischaemic heart disease and LDL 3.3 on atorvastatin 20mg daily.<br>Outcome: Dose increased to 80mg	EXTREME	UNLIKELY	**HIGH**	3.1
Oxybutynin not charted. Outcome: RMO added.	MINOR	POSSIBLE	**MEDIUM**	1.6
Prn oral oxycodone, no paracetamol order.<br>Outcome: Regular paracetamol charted	MINOR	UNLIKELY	**MEDIUM**	1.4
Non LAM: fosinopril swapped to Ramipril	NEGLIGIBLE	RARE	**LOW**	6.2
Isosorbide mononitrate doses *bd*. May cause tolerance.<br>Outcome: Changed to daily dose	MODERATE	LIKELY	**HIGH**	3.2
Patient taking simvastatin 40mg, IHD. Started on voriconazole 200mg bd.<br>Outcome: Dr withheld simvastatin while on voriconazole to prevent rhabdo.	EXTREME	POSSIBLE	**HIGH**	1.3
Atorvastatin 40mg charted, usual dose 10mg. Unintentional error.<br>Outcome: Dr changed to usual dose	MINOR	RARE	**LOW**	3.2

KPI cheatsheet - DRP #1

APOLOGIES THIS IS NOT COMPLETELY IN NUMERICAL ORDER - IT WOULDN'T FIT ON THE DATA COLLECTION SHEET!!!

DRP	FIELD	DEFINITION
1.4	**Prescription:** Indication not treated	The patient has a condition which requires drug therapy, which is not being treated. A medical condition requires the initiation of drug therapy, there is a need for additional drug, or there is an untreated medical condition. Preventive drug therapy is required to reduce the risk of developing a new condition.
1.2	No indication for drug	There is no therapeutic reason for the patient to be prescribed this drug. Indication does not warrant drug treatment, or medication condition is more appropriately treated with no drug. Drug therapy is being taken to treat an avoidable adverse drug reaction associated with another medication.
1.3	Interaction present	A drug/drug, drug/food or drug/alcohol interaction has been identified. This inappropriate combination may or may not cause toxicity. An undesirable reaction may be caused by increasing/ decreasing the effect of a drug, and may cause or causes a hypersensitivity/toxic reaction.
1.1	Inappropriate drug (C/I, not optimal drug)	Drug is contraindicated due to condition or comorbidity, or is not the best drug for the patient's treatment. Inappropriate drug due to contraindication, ineffectiveness, regimen (regular rather than "PRN") or safer alternative available. An unnecessary drug is taken because of the use of another drug. Drug is not effective for the indication being treated, medical condition is refractory to drug or there are safer and more effective alternatives. Inappropriate duplication of therapeutic group or active ingredient.
1.5	More cost-effective drug available	The medication is not the approved cost-effective hospital medication, the medication is not on the PBS, costing more out of pocket expenses for the patient The drug is not the most effective for the medical condition, and a different drug is needed. Preferred formulary alternative, OTC alternative and generic alternative is available. The drug is available in a combination dosage form but was given separately.
1.6	Omission (medication not charted)	The patient's usual medication has not been charted, and this is not clinically appropriate.
1.7	Duplication (drug doubled up)	Too many drugs prescribed for indication. Multiple drug products are being used for a condition that requires single drug therapy.
2.1	**Drug form** inappropriate/ suboptimal	The drug form is not appropriate for the patient. Inappropriate drug form may cause adherence or cost issues. May arise from a prescribing error or a dispensing error.
3.1	**Dose** too low	Drug dose is too low, including under-treatment. Based on pharmacokinetic or pharmacodynamic considerations, prescribed recommendations or previous dose, the dose is too low to produce the desired response. May result from prescribing error or dispensing error.
3.2	Dose too high	Drug dose is too high, which may or may not cause toxicity. Includes prescribed dose too high based on error, reference dose ranges, according to previous therapy, and according to a particular parameter of the patient such as renal function.
3.3	Dose not frequent enough	Dosage regimen not frequent enough, and the condition is thus undertreated. The dosage interval is too infrequent to produce the desired response. Sub-optimal dosing scheme may be due to prescriber intent or error.
3.4	Dose too frequent	Dosage regimen too frequent, or dosage interval is too short for the patient, whether due to prescriber intent or error.
3.6	Unclear/incomplete/ illegible instructions	It is not apparent what the instructions say, or they are not fully specified. The nurses cannot read the drug order on the chart, or The patient or person who is administering cannot or does not understand the instructions or how to properly take or use the drug product and dosage regimen.
4.1	Treatment **duration**	The treatment has not been charted for the full duration required. Duration of treatment is too short to produce the desired effect. (Or treatment duration has not been specified when it should be e.g. antibiotics)
4.2	Duration too long	Duration of treatment is longer than therapeutically necessary for this patient.

KPI cheatsheet - DRP #2

APOLOGIES THIS IS NOT COMPLETELY IN NUMERICAL ORDER - IT WOULDN'T FIT ON THE DATA COLLECTION SHEET!!!

DRP	FIELD	DEFINITION
5.4	**Administration:** Drug not administered	The medication was not administered, whether intentionally or accidentally: the patient chose to discontinue a drug by choice or for an illogical or irrational reason, or the patient forgot to take the drug, or the drug is too expensive for the patient.
5.1	Inappropriate administration timing/ dosing intervals	Inappropriate timing of administration and/or dosing intervals by patient, carer or nurse. This includes taking too little, too much, erratic use of medicine, the dosage regimen administered or changed too rapidly; the drug dosage was administered or escalated too rapidly resulting in an adverse reaction. Suboptimal dosing scheme, whether administered by health personnel or patient.
5.2	Underadministration of drug	Drug underused/underadministered, accidentally or intentionally. The patient chose, or the carer caused the patient, to take the wrong dose, which was lower than prescribed, or on a "when required basis" rather than regularly, for whatever reasons (misunderstanding, feeling better/worse, fear of adverse effects, did not believe the drug was effective/believed drug was toxic, occasionally forgot to take drug, regimen complex, underadministered by health care professional/carer).
5.3	Overadministration of drug	Whether accidentally or intentionally, the patient chose, or the carer caused the patient, to take the wrong dose, which was higher than prescribed, for whatever reasons (misunderstanding of directions, forgot they had already taken the drug, or overadministered/administered in error).
5.5	Wrong drug selected from shelf, taken or administered	Whether through ignorance or poor memory, the wrong drug is taken or administered. The pharmacist selects the wrong/expired drug from the shelf (dispensing error), the nurse administers drug from the wrong patient's chart, or the patient takes someone else's drug.
5.6	Drug abused	Intentional unregulated overuse of a drug for a non-indicated purpose, including over-the-counter drugs. This can include drug abuse, alcohol use or smoking.
5.7	Drug/form not used as directed	Patient, carer or nurse unable to use or does not use drug form as directed. This may be due to difficulty or ignorance. The patient is not able to swallow or administer the drug therapy as intended, or cannot self-administer the drug product appropriately. The drug was not administered by the appropriate route or method, or patient/carer does not understand how to properly take or use the drug product and dosage regimen.
5.8	Administration information needed to be given	Adequate information about drug not provided, incorrect information about drug or disease state was provided. Inadequate information about disease state management not provided, understood or followed. The nurse/carer/patient requires further information in order to have correct information about drug, disease state or drug order.
5.9	Inappropriate storage	Patient or nurse stores drug inappropriately, which may cause loss of potency or inability to locate stock when needed for administration (e.g. eye drops stored in the fridge when not needed, the next nurse can't find them overnight and writes N (for no supply) on chart) and/or stockpiling of medications.
6.1	Logistics: error in drug selection	The prescriber selected the incorrect medication but intended to select a different medication on an electronic medication record system. This is an accidental error on the part of the prescriber.
6.2	Drug unavailable (non-LAM/PBS, OOS)	The patient/carer had/will have difficulties obtaining the drug, or a drug has been discontinued, is not on formulary or is out of stock. The drug product is not available for the patient, or sufficient supply of the drug is not available to the patient.
6.3	Illegal, or incomplete Rx order	A drug order does not meet legislative requirements, , incomplete, or discrepant. The drug order may have no directions, is missing necessary information / signature, there is a lack of documentation of the drug chart/prescription.
7.1	Disease state monitoring or TDM required	Monitoring of drug or disease state should be done Eg BGL, drug level, bp, HR, ECG
7.3	Inappropriate test ordered	Monitoring of drug or disease state is inappropriate, or is too frequent to be appropriate, (or test taken at the incorrect time?)
8.1	Known ADR, including allergy/previous ADR	A patient is prescribed or administered a medicine to which they have had a previously documented Allergy or ADR
9.1	Therapeutic advice	Medicine consultation to treating team and medication information
	Clear spaces	Use these spaces when there is insufficient boxes for a DRP, or an intervention does not fit in the above categories
